# Streptococcus suivaginalis sp. nov., Streptococcus iners sp. nov. and Streptococcus iners subsp. hyiners subsp. nov. isolated from pigs

**DOI:** 10.1099/ijsem.0.006631

**Published:** 2025-01-22

**Authors:** Tracy L. Nicholson, Keira L. Stuart, Darrell O. Bayles

**Affiliations:** 1 National Animal Disease Center, Agricultural Research Service, USDA, Ames, IA, USA; 2 National Veterinary Services Laboratories, Animal Plant Health Inspection Service, USDA, Ames, IA, USA

**Keywords:** microbiome, new taxon, *Streptococcus*, swine, veterinary microbiology

## Abstract

Three novel strains within the genus *Streptococcus* (29887^T^, 29892^T^ and 29896^T^) were isolated from healthy pigs during routine veterinary physical exams. All three strains were non-motile and non-spore-forming Gram-positive cocci. The complete genome of each strain was attained, and phylogenetic analyses were performed. Comparison of the genomes of 29887^T^, 29892^T^ and 29896^T^ to the genomes of other *Streptococcus* strains revealed digital DNA–DNA hybridization (dDDH) values between 21.2% and 53.9% and average nucleotide identity (ANI) values between 70.00% and 94.44%. Phylogenetic analyses suggested that each strain, 29896^T^ (*S. suivaginalis* sp. nov.), 29887^T^ (*S. iners* sp. nov.) and 29892^T^ (*S. iners* subsp. *hyiners* subsp. nov.), may represent a novel species within the genus *Streptococcus*, while ANI analysis indicated that strains 29896^T^ (*S. suivaginalis* sp. nov.) and 29887^T^ (*S. iners* sp. nov.) represent novel species within the genus *Streptococcus,* and 29892^T^ (*S. iners* subsp. *hyiners* subsp. nov.) represents a novel subspecies of 29887^T^ (*S. iners* sp. nov.). Based upon the combined data presented in this study, two novel species, *Streptococcus suivaginalis* sp. nov. (type strain, 29896^T^=NRRL B-65677^T^=NCTC 14941^T^) and *Streptococcus iners* sp. nov. (type strain, 29887^T^=NRRL B-65675^T^=NCTC 14939^T^) are proposed, and one novel subspecies, *Streptococcus iners* subsp. *hyiners* subsp. nov. (type strain, 29892^T^=NRRL B-65676^T^=NCTC 14940^T^) is proposed.

## Introduction

The genus *Streptococcus* represents a large heterogeneous group of Gram-positive, catalase-negative and non-motile cocci (https://lpsn.dsmz.de/genus/streptococcus). This taxon has undergone substantial expansion and revision, with novel species continually being isolated from humans, animals and environmental samples. *Streptococcus* species have been implicated as causative agents of numerous human diseases including pneumonia, endocarditis, meningitis, otitis media, impetigo and urinary tract infections [1,4]. However, most streptococci are commensals that are part of the normal microbiota of the skin, alimentary, respiratory and genitourinary tracts of humans and various warm-blooded animals [3,5]. In this article, we report the phenotypic and phylogenetic characterization of three strains of unusual *Streptococcus*-like organisms isolated from vaginal swabs obtained from healthy pigs that were void of any observable clinical signs of disease or lesions during routine veterinary physical exams. Based on the data presented, two novel species and one novel subspecies of the genus *Streptococcus* are described.

## Isolation and ecology

Samples collected from the vaginas of pigs (using sterile swabs during veterinary physical exams at a swine facility in Ohio) were submitted by clients to the University of Minnesota Veterinary Diagnostic Laboratory. Swabs were inoculated onto tryptic soy agar (TSA) containing 5% defibrinated sheep blood and incubated at 37 °C in ambient air supplemented with 5±1% CO_2_ (henceforth referred to as 5% CO_2_ atmosphere). Bacterial growth on plates was visibly observed after 1–2 days. Individual colonies from the agar plates were sub-cultivated to generate pure bacterial cultures. All strains were routinely sub-cultured and grew well on either TSA with 5% sheep blood, Columbia agar with 5% sheep blood or Todd–Hewitt broth supplemented with 0.2% yeast extract (THY) +5% filtered heat-inactivated horse serum in 5% CO_2_ atmosphere. Bacterial stocks were saved in Columbia broth supplemented with 15% (v/v) glycerol or horse serum at −80 °C.

## Genome features

Genomic DNA was extracted using previously described methods [6]. The Illumina DNA PCR-Free Library Prep Kit (Illumina) was used for library creation and sequenced on a HiSeq 3000 instrument, generating 2×150 bp paired-end reads. Illumina data were assessed for quality using FastQC (https://www.bioinformatics.babraham.ac.uk/projects/fastqc/), and BBduk was used for adapter trimming (https://sourceforge.net/projects/bbmap/). For long-read PacBio sequencing, a HiFi Multiplex library with a 10–20 kb insert size was prepared and sequenced on a Sequel II system using an SMRT Cell 8M in CCS/HiFi mode. PacBio CCS reads were assembled using Flye v. 2.9.1 in --pacbio-hifi mode with options --asm-coverage 100 --g 2.2 m [7]. The resulting long-read assemblies were closed circular chromosomes and a closed circular plasmid for strain 29887^T^ (*S. iners* sp. nov.), which were then error-corrected using Pilon v. 1.23 [8] and the Illumina data. Unless otherwise specified, default software parameters were used. The final chromosome assemblies were oriented to begin at the *dnaA* gene. The assemblies were then annotated using the NCBI Prokaryotic Genome Annotation Pipeline v. 6.4 [9]. Assembly statistics and accession numbers are summarized in Table 1.

**Table 1.** Summary of sequenced 29887^T^ (*S. iners* sp. nov.), 29892^T^ (*S. iners* subsp. *hyiners* subsp. nov.) and 29896^T^ (*S. suivaginalis* sp. nov.)

	29887^T^*S. iners* sp. nov.	29892^T^*S. iners* subsp. *hyiners* subsp. nov.	29896^T^*S. suivaginalis* sp. nov.
ChromosomeSize (bp)	2,304,690	2,055,713	2,133,007
G+C content in mol%	42.1	42.5	43.4
Predicted CDSs	2165	1993	1896
rRNA(16 S-23S-5S)	4-4-4	4-4-4	4-4-4
tRNA	58	55	56
ncRNA	3	3	3
tmRNA	1	1	1
CRISPR/Cas	Yes	No	Yes
Plasmid	1	0	0
Plasmid size (bp)	10,787	na	na
Predicted plasmid CDSs	15	na	na
Accession numbers*	CP118735CP118736	CP118734	CP118733

na, Not applicable due to the absence of plasmid.

* Accession numbers provided are for chromosome followed by plasmid.

## Phylogeny

To establish the phylogenetic relationship of strains 29887^T^ (*S. iners* sp. nov.), 29892^T^ (*S. iners* subsp. *hyiners* subsp. nov.) and 29896^T^ (*S. suivaginalis* sp. nov.) to other *Streptococcus* species, 16S rRNA and *recN* gene [10] sequence analyses were carried out. The RefSeq-annotated versions of all *Streptococcus* sp. representative genomes were retrieved from NCBI on 2023-07-11. At the time of download, there were 114 representative *Streptococcus* sp. genomes. The DNA sequences corresponding to the *recN* and 16S rRNA gene sequences were extracted from the annotation files and the genome sequences of 29887^T^ (*S. iners* sp. nov.), 29892^T^ (*S. iners* subsp. *hyiners* subsp. nov.) and 29896 ^T^ (S. *suivaginalis* sp. nov.). Since some of the representative genomes were drafts, any partial *recN* or partial 16S rRNA gene sequences were excluded from the analyses. Additionally, for 16S rRNA gene sequences, the multiple 16S rRNA gene sequences within each genome were constrained to a single copy of each variant found within the genome. The DNA sequences were aligned using the MAFFT v. 7.505 G-INS-I algorithm with default parameters [11]. The aligned sequences were further curated with Gblocks v. 0.91b, implementing relaxed stringency with the following parameters: (-t=d -b2=60 -b4=4 -b5=all) [12]. Phylogenetic model testing on the aligned sequences was performed with the Phangorn v. 2.11.1 modelTest package [13]. A distance matrix was computed using the Phangorn dist.ml function, specifying the JC69 model. Trees were estimated from the distance matrix using the Phangorn neighbour-joining (NJ) and unweighted pair group with the arithmetic mean method. The parsimony scores for both *recN* and 16S trees indicated the NJ-estimated trees were the most parsimonious. The likelihood of the trees was computed using the Phangorn pml method and the NJ trees. This was subsequently optimized using the Phangorn optim.pml algorithm with the parameters ‘rearrangement=‘stochastic’, optGamma=TRUE, optInv=TRUE and model='GTR'’ parameters. Non-parametric bootstrap analysis was carried out using the Phangorn bootstrap.pml method. Midpoint trees were plotted using the Phangorn plotBS method, and the plotted trees were printed using the Bioconductor ggtree package [14].

**Fig. 1.**
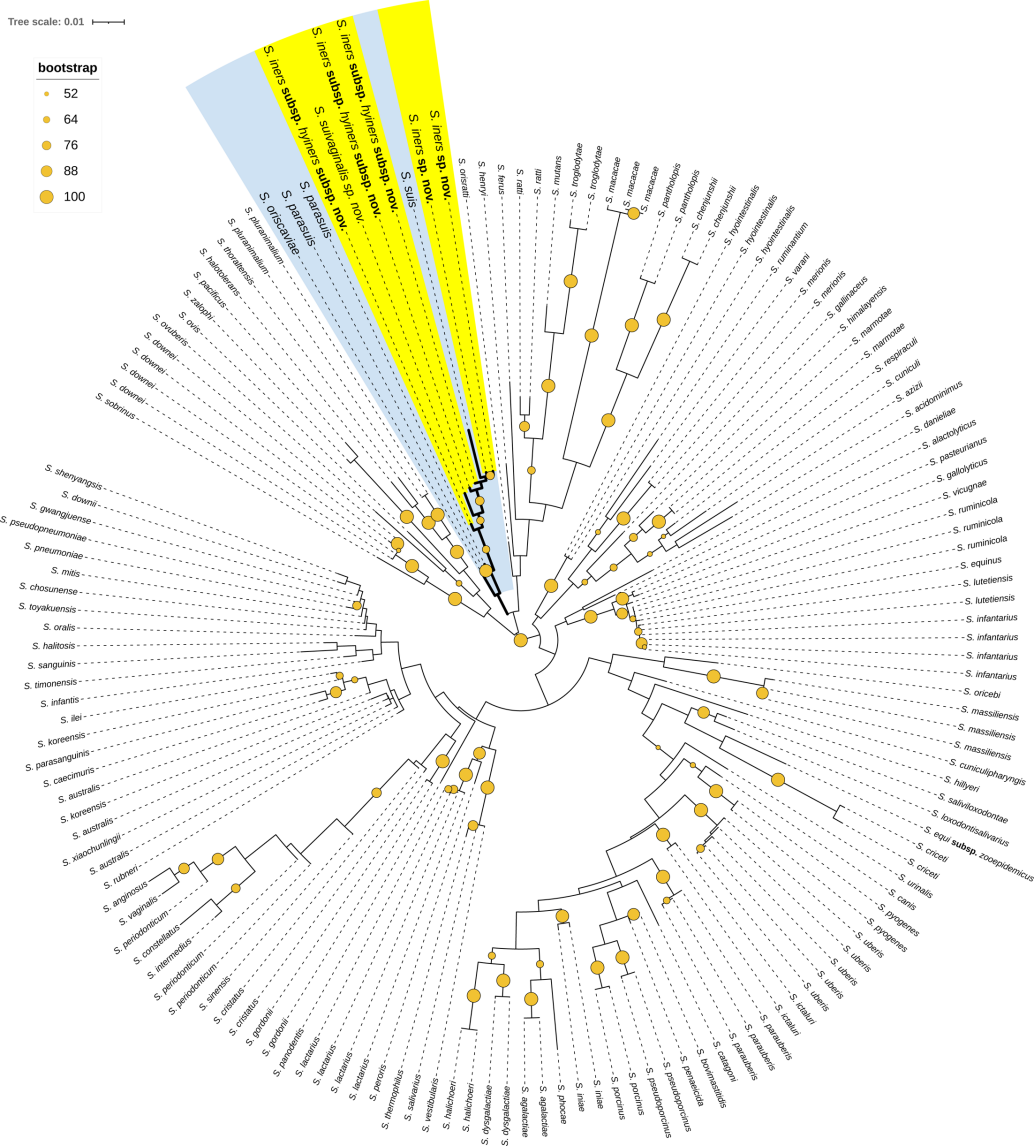
Phylogenetic tree inferred from 16S rRNA gene sequence comparison using the NJ method, showing the relationships of *S. iners* sp. nov., *S. iners* subsp. *hyiners* subsp. nov., *S. suivaginalis* sp. nov. with other *Streptococcus* species. Bootstrap values (expressed as percentages of 100 replications) >50 are shown at branching points. Bar, 1% sequence divergence. Clade comprising the novel strains is highlighted in blue. All strains and their corresponding names, GenBank designations, locus-tag prefixes and RefSeq accession numbers are listed in Table S1 available in the online Supplementary Material.

**Fig. 2.**
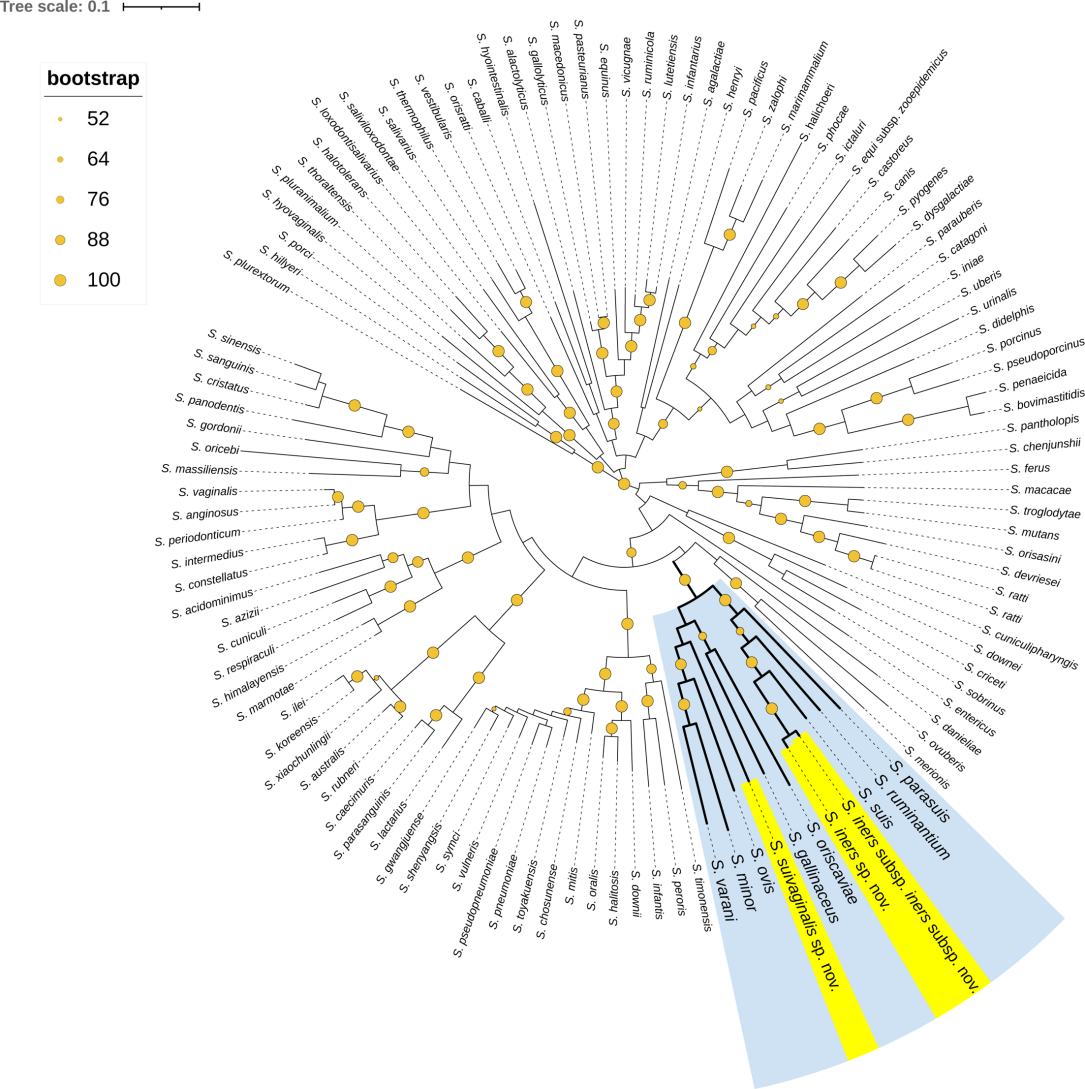
Phylogenetic tree inferred from *recN* gene sequence comparison using the NJ method, showing the relationships of *S. iners* sp. nov., *S. iners* subsp. *hyiners* subsp. nov., and *S. suivaginalis* sp. nov. with other *Streptococcus* species. Bootstrap values (expressed as percentages of 100 replications) >50 are shown at branching points. Bar, 1% sequence divergence. Clade comprising the novel strains is highlighted in blue and novel strains are highlighted in yellow. All strains and their corresponding names, GenBank designations, locus-tag prefixes and RefSeq accession numbers are listed in Table S1.

The phylogenetic tree based on the 16S rRNA gene sequences affirmed that each novel species belonged within the genus of *Streptococcus* and placed each novel species as a separate branch within a clade that included *S. suis*. This clade, which included strains 29887^T^ (*S. iners* sp. nov.), 29892^T^ (*S. iners* subsp. *hyiners* subsp. nov.) and 29896^T^ (*S. suivaginalis* sp. nov.) and *S. suis,* branched from *S. parasuis* (Fig. 1). Phylogenetic analysis based on the *recN* gene has been described as the best taxonomic tool for the identification of *Streptococcus* sp. by previous studies [10,15]. In the phylogenetic tree based on *recN* gene sequences, strains 29887^T^ (*S. iners* sp. nov.) and 29892^T^ (*S. iners* subsp. *hyiners* subsp. nov.) were observed to form separate and distinct branches from *S. suis* within a clade that included *S. parasuis* and *S. ruminantium* (Fig. 2). Strain 29896^T^ (*S. suivaginalis* sp. nov.) was observed to form a separate and distinct branch within a different clade from 29887^T^ (*S. iners* sp. nov.), 29892^T^ (*S. iners* subsp. *hyiners* subsp. nov.) and *S. suis*, which included *S. oriscaviae, S. gallinaceus, S. orvis, S. minor and S. varani* (Fig. 2). Combined, these data suggest that each strain 29887^T^ (*S. iners* sp. nov.), 29892^T^ (*S. iners* subsp. *hyiners* subsp. nov.) and 29896^T^ (*S. suivaginalis* sp. nov.) may represent a novel species within the genus *Streptococcus*.

To further evaluate whether strains 29887^T^ (*S. iners* sp. nov.), 29892^T^ (*S. iners* subsp. *hyiners* subsp. nov.) and 29896^T^ (*S. suivaginalis* sp. nov.) represent a novel species within the genus *Streptococcus,* genome sequence data for each novel strain were uploaded to the Type (Strain) Genome Server (TYGS) (https://tygs.dsmz.de; accessed 2024-04-03) for 16S rRNA gene sequence and whole-genome-based phylogenetic analyses, including digital DNA–DNA hybridization (dDDH) [16,17]. The determination of the closest related type strains was determined by the TYGS using the lowest 16S rRNA gene sequence distance from all type strain genomes available in the TYGS database to each novel strain, as well as the lowest *k*-mer-based MaSH distance from all type strain genomes in the TYGS database to each novel strain [18,19]. The resulting genome blast distance phylogeny (GBDP) distances were used to infer balanced minimum-evolution trees via FastME 2.1.6.1 [20]. Branch support was inferred from 100 pseudo-bootstrap replicates each. The trees were rooted at the midpoint [21] and visualized with iTOL [22].

**Fig. 3.**
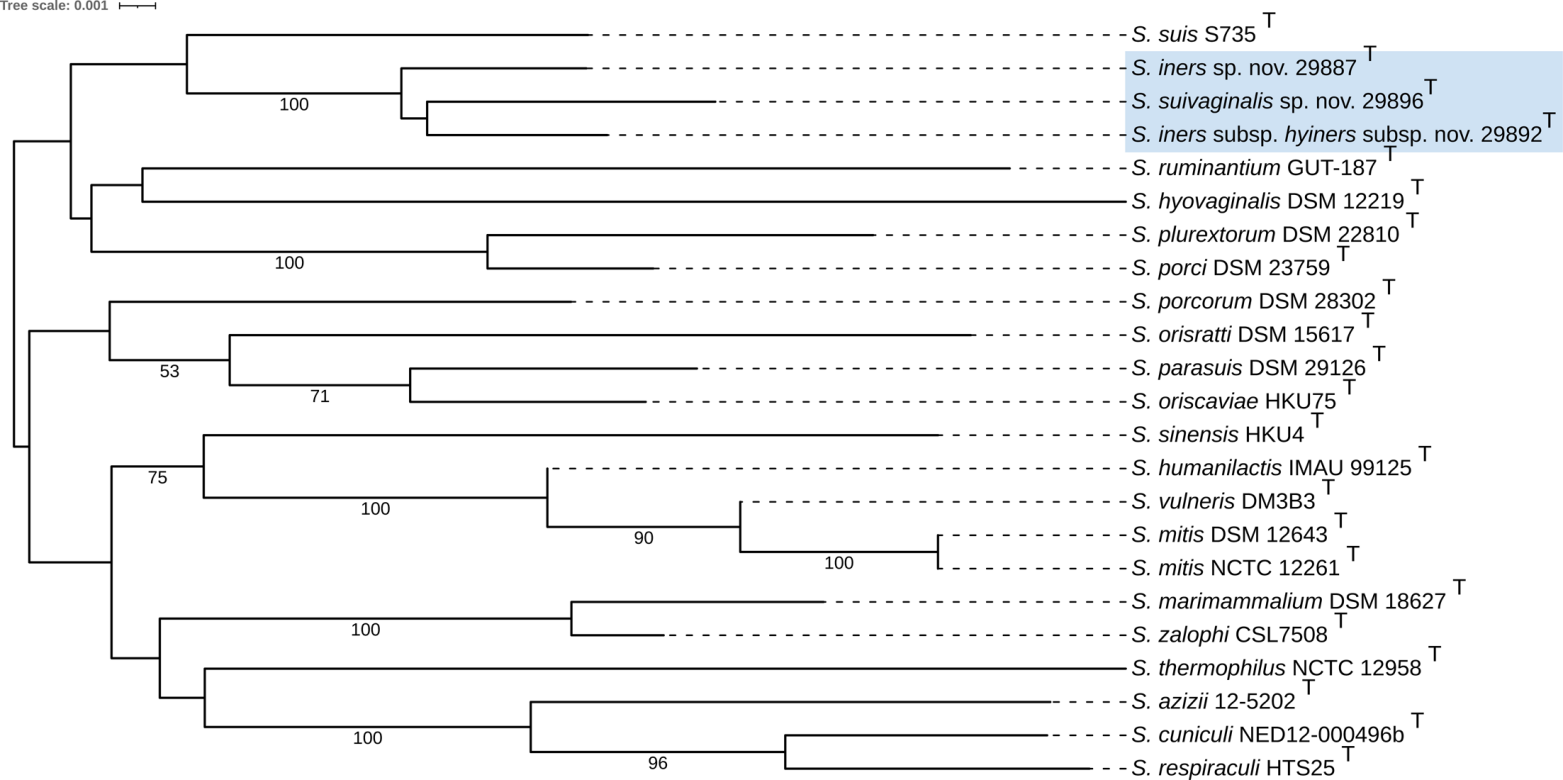
16S rRNA gene-based phylogeny as provided by TYGS [16]. Tree inferred with FastME 2.1.6.1(20) from GBDP distances calculated from 16S rDNA gene sequences. The branch lengths are scaled in terms of the GBDP distance formula *d*_5_. The numbers below branches are GBDP pseudo-bootstrap support values >60% from 100 replications. The tree was rooted at the midpoint [21] and visualized with iTOL [22]. Novel strains are highlighted in blue.

**Fig. 4.**
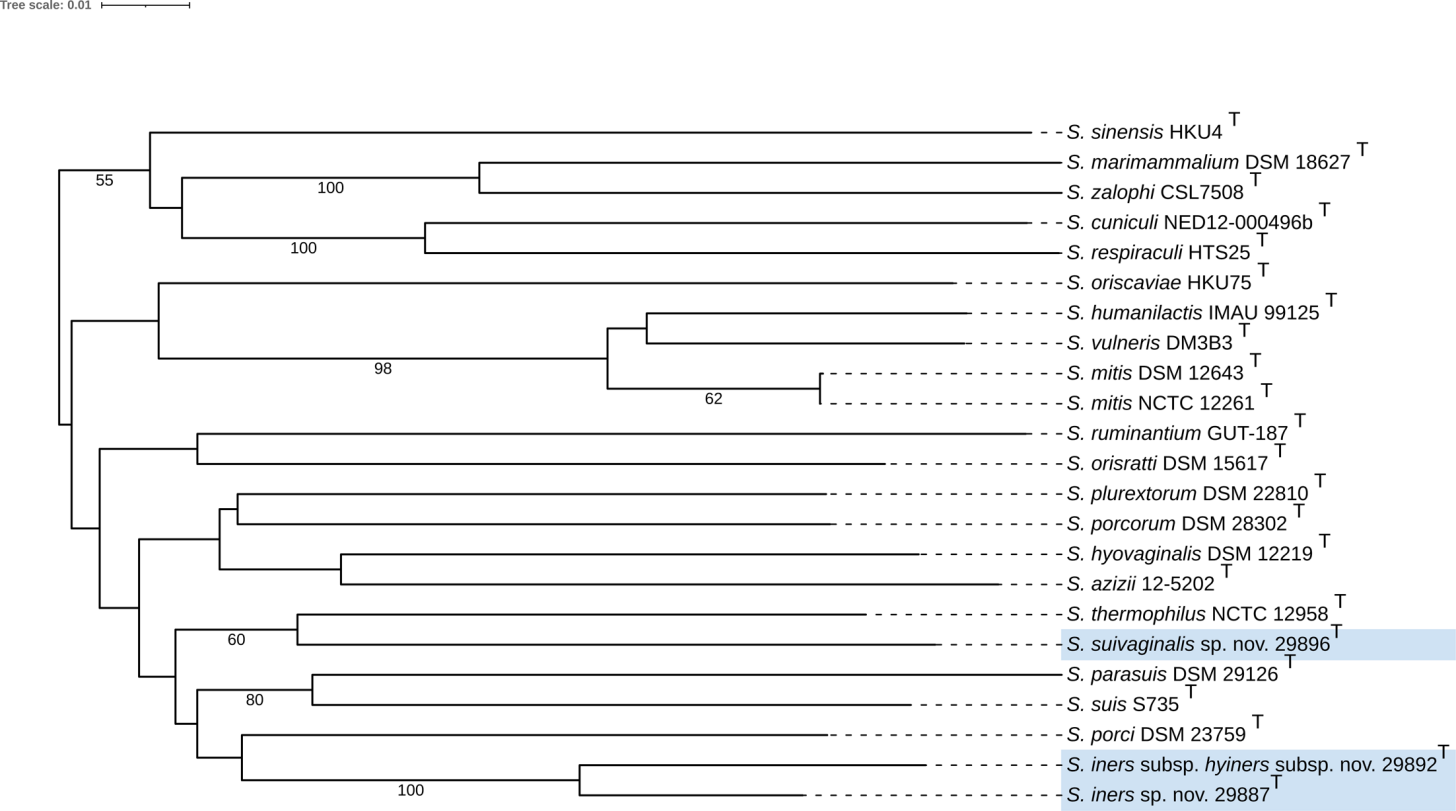
Whole-genome sequence-based phylogeny as provided by TYGS [16]. Tree inferred with FastME 2.1.6.1(20) from GBDP distances calculated from genome sequences. The branch lengths are scaled in terms of GBDP distance formula *d*_5_. The numbers below branches are GBDP pseudo-bootstrap support values >60% from 100 replications. The tree was rooted at the midpoint [21] and visualized with iTOL [22]. Novel strains are highlighted in blue.

The phylogenetic tree provided by TYGS, based on 16S rRNA gene sequences, confirmed that each novel strain belonged within the genus of *Streptococcus* and placed each novel strain as a separate branch within a clade that included *S. suis* (Fig. 3). In the phylogenetic tree based on whole-genome sequences, strains 29887^T^ (*S. iners* sp. nov.) and 29892^T^ (*S. iners* subsp. *hyiners* subsp. nov.) were observed to form separate and distinct branches within a clade that included *S. suis* and *S. porci* (Fig. 4). Strain 29896^T^ (*S. suivaginalis* sp. nov.) was observed to form a separate and distinct branch within a different clade from 29887^T^ (*S. iners* sp. nov.) and 29892^T^ (*S. iners* subsp. *hyiners* subsp. nov.), which included *S. thermophilus* (Fig. 4). Combined, the data provided by the TYGS suggest that each strain 29896^T^ (*S. suivaginalis* sp. nov.), 29887^T^ (*S. iners* sp. nov.) and 29892^T^ (*S. iners* subsp. *hyiners* subsp. nov.), may represent a novel species within the genus *Streptococcus*.

Pairwise dDDH was determined using the TYGS and a type-based species clustering using a 70% dDDH radius around each of the closest type-strain genomes available in the TYGS [16]. Subspecies clustering was done using a 79% dDDH threshold [23]. The dDDH values for 29887^T^ (*S. iners* sp. nov.) compared to each of the closest type-strains ranged from 21.6% to 35.7%, and the closest related *Streptococcus* sp. to 29887^T^ (*S. iners* sp. nov.) was 29892^T^ (*S. iners* subsp. *hyiners* subsp. nov.) with a dDDH value of 53.9% (Table 2). The dDDH values for 29892^T^ (*S. iners* subsp. *hyiners* subsp. nov.) compared to each of the closest type-strains ranged from 21.2% to 33.3% (Table 2). The dDDH values for 29896^T^ (*S. suivaginalis* sp. nov.) compared to each of the closest type strains ranged from 20.8% to 33.1%, and the closest related *Streptococcus* sp. to 29896^T^ (*S. suivaginalis* sp. nov.) was * S. thermophilus* strain NCTC 12958 ^T^, with a dDDH value of 33.1% (Table 2). All of the dDDH values, including the dDDH value of 53.9% between 29887^T^ (*S. iners* sp. nov.) and 29892^T^ (*S. iners* subsp. *hyiners* subsp. nov.), were below the ≥70% dDDH required for prokaryotic species delineation [24,25].

**Table 2.** Pairwise dDDH values between the three novel species and the closest type-strain genomes available in the TYGS

Taxa*	*S. iners* sp. nov. 29887**^T^**			*S. iners* subsp. *hyiners* subsp. nov. 29892**^T^**			*S. suivaginalis* sp. nov. 29896**^T^**		
	dDDH†	C.I.‡	G+C§	dDDH	C.I.	G+C	dDDH	C.I.	G+C
*S. azizii* 12-5202^T^	25.3	[23.0–27.8]	0.69	24.1	[21.8–26.6]	0.25	22.7	[20.4–25.2]	0.59
*S. cuniculi* NED12-000496b^T^	21.6	[19.4–24.1]	1.37	21.2	[19.0–23.6]	0.93	20.8	[18.6–23.3]	0.09
*S. humanilactis* IMAU 99125^T^	24.3	[22.0–26.8]	2.07	26	[23.6–28.4]	2.51	24	[21.7–26.4]	3.35
*S. hyovaginalis* DSM 12219^T^	29.2	[26.9–31.7]	2.21	30.3	[27.9–32.8]	2.65	26.7	[24.3–29.2]	3.49
*S. marimammalium* DSM 18627^T^	23.5	[21.2–25.9]	8.89	22.8	[20.5–25.3]	9.33	26.1	[23.8–28.6]	10.17
*S. mitis* DSM 12643^T^	26	[23.7–28.5]	1.62	26.4	[24.0–28.9]	2.06	25.8	[23.5–28.3]	2.9
*S. oriscaviae* HKU75^T^	23.8	[21.5–26.3]	1.98	23.7	[21.4–26.1]	1.54	22.9	[20.7–25.4]	0.7
*S. orisratti* DSM 15617^T^	28.2	[25.8–30.7]	3.54	29.2	[26.8–31.7]	3.98	26.7	[24.3–29.2]	4.82
*S. parasuis* DSM 29126^T^	26.3	[24.0–28.8]	2.2	25.4	[23.1–27.9]	2.64	26.2	[23.9–28.7]	3.48
*S. plurextorum* DSM 22810^T^	32.3	[29.9–34.8]	0.95	32.1	[29.7–34.6]	1.39	29.8	[27.4–32.3]	2.23
*S. porci* DSM 23759^T^	35.7	[33.2–38.2]	1.31	30.2	[27.8–32.7]	1.75	28.9	[26.5–31.4]	2.59
*S. porcorum* DSM 28302^T^	30.4	[28.0–32.9]	1.31	32.6	[30.2–35.1]	4.33	28.9	[26.5–31.4]	5.17
*S. respiraculi* HTS25^T^	21.7	[19.4–24.1]	3.89	21.4	[19.2–23.8]	0.56	22.3	[20.1–24.8]	1.4
*S. ruminantium* GUT-187^T^	23.4	[21.1–25.9]	0.12	23.2	[20.9–25.7]	2.5	23	[20.7–25.5]	3.34
*S. sinensis* HKU4^T^	23.5	[21.2–25.9]	2.06	23.8	[21.5–26.3]	0.35	23.7	[21.4–26.1]	1.19
*S. suis* S735^T^	30.6	[28.2–33.1]	0.09	30.1	[27.8–32.6]	1.11	28.8	[26.5–31.3]	1.95
*S. thermophilus* NCTC 12958^T^	30.4	[28.0–32.9]	0.67	33.3	[30.9–35.8]	3.49	33.1	[30.7–35.6]	4.33
*S. vulneris* DM3B3^T^	24.1	[21.8–26.6]	3.05	24.8	[22.5–27.3]	2.45	25.3	[23.0–27.8]	3.29
*S. zalophi* CSL7508^T^	23.2	[20.9–25.7]	2.01	24.8	[22.5–27.3]	7.73	26.7	[24.3–29.2]	8.57
*S. suivaginalis* sp. nov. 29896^T^	28.4	[26.1–30.9]	1.28	27.9	[25.6–30.4]	0.84			
*S. iners* sp. nov. 29887^T^				53.9	[51.2–56.6]	0.44	28.4	[26.1–30.9]	1.28
*S. iners* subsp. *hyiners* subsp. nov. 29892^T^	53.9	[51.2–56.6]	0.44				27.9	[25.6–30.4]	0.84

a*Taxa listed by *Streptococcus* sp. followed by strain designation.

b†Pairwise dDDH values (%) among 29896T (*S. suivaginalis* sp. nov.), 29887T (*S. iners* sp. nov.), and 29892T (*S. iners* subsp. *hyiners* subsp. nov.), and the closest type-strain genomes available in the TYGS, calculated based on the formula *d*_4_, reflecting the proportion of sequence identity within the homologous parts of the compared genomes.

c‡Confidence intervals (C.I.) (%) for pairwise dDDH values.

d§G +C content difference (%).

Average nucleotide identity (ANI) for all *Streptococcus* sp. representative genomes retrieved from NCBI in April 2023, along with the genomes of strains 29887^T^ (*S. iners* sp. nov.), 29892^T^ (*S. iners* subsp. *hyiners* subsp. nov.) and 29896^T^ (*S. suivaginalis* sp. nov.) was calculated using FastANI, which uses an algorithm based on MinHash mapping to calculate pairwise genome-to-genome ANI values [26]. ANI values for all representative *Streptococcus* sp. with an ANI value of 80 or higher are shown in Table 3. The closest related *Streptococcus* sp. to 29887^T^ (*S. iners* sp. nov.) were 29892^T^ (*S. iners* subsp. *hyiners* subsp. nov.) with 94.24 ANI, * S. suis* BM407 with 87.28 ANI and 29896^T^ (*S. suivaginalis* sp. nov.) with 86.29 ANI (Table 3). The closest related *Streptococcus* sp. to 29892^T^ (*S. iners* subsp. *hyiners* subsp. nov.) were 29887^T^ (*S. iners* sp. nov.) with 94.44 ANI, *S. suis* BM407 with 87.23 ANI and 29896^T^ (*S. suivaginalis* sp. nov.) with 86.28 ANI (Table 3). The closest related *Streptococcus* sp. to 29896^T^ (*S. suivaginalis* sp. nov.) were *S. suis* BM407 with 87.10 ANI, 29887^T^ (*S. iners* sp. nov.) with 86.43 ANI and 29892^T^ (*S. iners* subsp. *hyiners* subsp. nov.) with 86.37 ANI. The closest representative *Streptococcus* sp. to any of the three novel strains were *S. suis* BM407 (GCA_000026745.1), followed by *S. parasuis* (GCA_021654455.1), *S. porci* (GCA_000423765.1) and *S. plurextorum* (GCA_000423745.1). While the ANI values between strains 29887^T^ (*S. iners* sp. nov.) and 29892^T^ (*S. iners* subsp. *hyiners* subsp. nov.) were below the suggested threshold ANI value of 95% for species delimitation, they were very close to this threshold value [25,27, 28]. Due to the ANI values of 94.24 and 94.44 between 29887^T^ (*S. iners* sp. nov.) and 29892^T^ (*S. iners* subsp. *hyiners* subsp. nov.), respectively, we propose 29892^T^ (*S. iners* subsp. *hyiners* subsp. nov.) is a subsp. of 29887^T^ (*S. iners* sp. nov.). Therefore, while the phylogenetic analyses (Figs1^4^) suggest that each strain 29896^T^ (*S. suivaginalis* sp. nov.), 29887^T^ (*S. iners* sp. nov.) and 29892^T^ (*S. iners* subsp. *hyiners* subsp. nov.) may represent a novel species within the genus *Streptococcus,* the ANI analysis indicates that strains 29896^T^ (*S. suivaginalis* sp. nov.) and 29887^T^ (*S. iners* sp. nov.) represent novel species within the genus *Streptococcus,* and 29892^T^ (*S. iners* subsp. *hyiners* subsp. nov.) represents a novel subspecies of 29887^T^ (*S. iners* sp. nov.).

**Table 3.** ANI values based on whole-genome sequences between the three novel species and closely related *Streptococcus* strains Strains: 1. 29887^T^ (*S. iners* sp. nov.; CP118735); 2. 29892^T^ (*S. iners* subsp. *hyiners* subsp. nov.; CP118734); 3. 29896^ T^ *S*. *suivaginalis* sp. no.; CP118733); 4. *S. suis* BM407* (GCF_000026745.1); 5. *S. parasuis* SUT-286^T^ (NZ_AP024276); 6. *S. plurextorum* DSM 22810^T^ (GCF_000423745.1); 7. *S. porci* DSM 23759^T^ (GCF_000423765.1); 8. *S. oriscaviae* HKU75^T^ (GCF_018137985.1); 9. *S. ruminantium* GUT187^T^ (GCF_003609975.1). Values that exceed the threshold for species delineation are indicated in bold. ANI values below the cutoff threshold for reporting (78%) by FastANI are indicated by nr.

Taxa	1	2	3	4	5	6	7	8	9
1	100	**94.44**	**86.43**	**87.42**	**84.42**	**84.02**	**83.66**	**80.62**	**80.36**
2	**94.24**	100	**86.37**	**87.04**	**83.90**	**83.79**	**82.28**	**81.17**	**80.02**
3	**86.29**	**86.28**	100	**86.72**	**84.66**	**83.23**	**82.16**	**80.40**	**79.92**
4	**87.28**	**87.23**	**87.10**	100	**86.95**	**83.66**	**83.14**	**80.67**	**80.96**
5	**84.51**	**84.27**	**84.41**	**87.09**	100	**82.03**	**nr**	**80.60**	**80.03**
6	**84.17**	**84.19**	**83.50**	**84.14**	**82.11**	100	**82.11**	**nr**	**nr**
7	**83.83**	**82.36**	**82.06**	**83.23**	**nr**	**82.32**	100	**nr**	**nr**
8	**80.68**	**81.19**	**80.42**	**80.92**	**80.41**	**nr**	**nr**	100	**79.11**
9	**80.29**	**80.47**	**80.05**	**80.89**	**79.98**	**nr**	**nr**	**79.00**	100
G+C content in mol%	42.1	42.5	43.4	41.1	40.0	41.1	40.8	44.1	40.0

nr, Not reported.

* NCBI RefSeq Genome or reference genome sequence for *S. suis*.

## Morphology and cultural properties

Cell morphology was observed using the conventional Gram stain procedure (Gram-Staining Kit, BD), and motility was examined by inoculation in a semisolid Columbia agar. Catalase activity was detected by the formation of bubbles in 3% (v/v) H_2_O_2_ solution. An oxidase activity test was performed using Remel BactiDrop Oxidase (Thermo Fisher Scientific). All strains were Gram-positive, catalase-negative, oxidase-negative, non-motile and non-sporulating cocci. Strain 29887^T^ (*S. iners* sp. nov.) presented in pairs or chains usually 10 or more cells long. Strain 29892^T^ (*S. iners* subsp. *hyiners* subsp. nov.) presented in pairs or small chains of approximately three to six cells in length. Strain 29896^T^ (*S. suivaginalis* sp. nov.) presented in pairs and small clusters. All three strains grew well in THY +5% filtered heat-inactivated horse serum, Tryptic soy broth (TSB) and Thioglycolate broth (THIO) at 37 °C in ambient air or 5% CO_2_ atmosphere during 24–48 h. In all broths, under optimal conditions (see below), the strains grew planktonically with moderate turbidity and moderate sediment observed.

TSB was used to determine the optimal temperature range for the growth of the strains in either ambient air or anaerobic conditions (GasPak EZ anaerobe pouch system; BD). All strains grew well at temperatures 30–42 °C in both ambient air and anaerobic conditions, with optimal growth observed between 35 and 38 °C. NaCl tolerance was tested at a concentration of TSB +6.5% (w/v), and no growth was observed for all strains. Colonies from growth on either TSA with 5% sheep blood or Columbia agar with 5% sheep blood were approximately 1 mm in diameter, round, non-pigmented and smooth with zones of *α*-haemolysis beginning to appear after 24 h. After 72 h, the zones of *α*-haemolysis became more prominent, measuring approximately 1–2 mm.

## Physiology and chemotaxonomy

API Rapid ID32 Strep (bioMérieux) was used to biochemically characterize the strains, and the StrepPRO Streptococcal Grouping Kit (Hardy Diagnostics) was used for Lancefield grouping. All three strains tested negative for arginine hydrolysis, Voges–Proskauer test and Hippurate hydrolysis, and the production of *β*-glucosidase, *α*-galactosidase, alkaline phosphatase, *β*-galactosidase, pyroglutamic acid arylamidase, *N*-acetyl-*β*-glucosaminidase, glycyl-tryptophan arylamidase, *β*-mannosidase and urease (Table 4). All three strains tested negative for the production of acid from methyl-*β*-d-glucopyranoside, tagatose, ribose, sorbitol, l-arabinose, d-arabitol, cyclodextrin, melibiose and melezitose (Table 4). Strain 29887^T^ (*S. iners* sp. nov.) was positive for Lancefield group G and positive for the production of *β*-glucuronidase, alanyl–pheylalanyl–proline–arylamidase and the production of acid from trehalose and sucrose. Strain 29892^T^ (*S. iners* subsp. *hyiners* subsp. nov.) was negative for Lancefield group A, B, C, D, F and G and was positive for the production of alanyl–pheylalanyl–proline–arylamidase and the production of acid from mannitol, lactose, trehalose, raffinose, maltose and sucrose (Table 4). Strain 29896^T^ (*S. suivaginalis* sp. nov.) was negative for Lancefield group A, B, C, D, F and G and positive for the production of *β*-galactosidase, alanyl–pheylalanyl–proline–arylamidase and the production of acid from mannitol, lactose, glycogen, pullulan, maltose and sucrose (Table 4).

**Table 4.** Differential phenotypic characteristics between the novel strains 29887^T^ (*S. iners* sp. nov.), 29892^T^ (*S. iners* subsp. *hyiners* subsp. nov.) and 29896^T^ (*S. suivaginalis* sp. nov.) and closely related type strains Strains 1. 29887^T^ (*S. iners* sp. nov.; CP118735); 2. 29892^T^ (*S. iners* subsp. *hyiners* subsp. nov.; CP118734); 3. 29896^T^ (*S. suivaginalis* sp. no.; CP118733); 4. *S. suis* S735^T^; 5. *S. parasuis* SUT-286^T^; 6. *S. plurextorum* 1956-02^T^; 7. *S. porci* 2923-03^T^; 8. *S. oriscaviae* HKU75^T^; 9. *S. ruminantium* GUT187^T^. Phenotypic data from this study and [33,36]. +, positive; −, negative; nr, not reported.

Characteristic	1	2	3	4	5	6	7	8	9
Production of:									
*β*-Glucosidase	−	−	−	+	−	−	−	−	+
*β*-Galactosidase-gar	−	−	+	+	−	+	+	−	−
*β*-Galactosidase-gal	−	−	−	+	−	+	−	−	−
*β*-Glucuronidase	+	−	−	−	−	+	−	−	−
*α*-Galactosidase	−	−	−	+	−	+	+	−	−
Alanyl–pheylalanyl–proline–arylamidase	+	+	+	+	+	+	+	−	+
Glycyl–tryptophan arylamidase	−	−	−	+	−	+	+	nr	+
Production of acid from:									
Mannitol	−	+	+	−	−	−	−	−	−
Lactose	−	+	+	+	+	+	+	+	+
Trehalose	+	+	−	+	+	+	+	+	+
Raffinose	−	+	−	+	+	−	−	−	−
l-Arabinose	−	−	−	+	−	−	−	−	−
Glycogen	−	−	+	+	+	−	+	+	+
Pullulan	−	−	+	+	+	−	+	−	+
Maltose	−	+	+	+	+	+	+	+	+

nr, Not reported.

## Antimicrobial activity

Phenotypic antibiotic resistance was determined using the broth microdilution method by National Veterinary Services Laboratories (Ames, IA), following standard operating procedures. Minimum inhibitory concentrations (MICs) were determined for each isolate using the Sensititre BOPO7F Plate (Thermo Fisher Scientific), with *Streptococcus pneumoniae* ATCC 49619 and *Mannheimia haemolytica* ATCC 33369 (ATCC, Manassas, VA) serving as quality control strains. MICs were evaluated in accordance with Clinical Laboratory Standards Institute (CLSI) recommendations, based on standards for resistance interpretations [29,30]. Strain 29887^T^ (*S. iners* sp. nov.) exhibited phenotypic resistance to tetracyclines, macrolide/lincosamide/streptogramin (MLSb), sulphonamides and pleuromutilin. Strain 29892^T^ (*S. iners* subsp. *hyiners* subsp. nov.) exhibited phenotypic resistance to beta-lactam/ penicillin, tetracyclines, MLSb and sulphonamides. Strain 29896^T^ (*S. suivaginalis* sp. nov.) exhibited phenotypic resistance to tetracyclines and MLSb.

Draft genomes were screened for chromosomal mutations and genes conferring antimicrobial resistance using ABRicate (Seeman T, Abricate, GitHub; http://github.com/tseemann/abricate) to identify antimicrobial resistance genes using the AMR gene databases from the Comprehensive Antibiotic Resistance Database (CARD) [31], ResFinder (Center for Genomic Epidemiology [32]) and the NCBI Bacterial Antimicrobial Resistance Reference Gene Database (BioProject Accession PRJNA313047), which was downloaded in April 2019. A minimum percent identity threshold of 80% was used to identify AMR genes in the assembled genomes. Strain 29887^T^ (*S. iners* sp. nov.) harboured a *tetO* and two *ermB* genes. Strain 29892^T^ (*S. iners* subsp. *hyiners* subsp. nov.) harboured *tetO*, *tetM* and two *ermB* genes. Strain 29896^T^ (*S. suivaginalis* sp. nov.) harboured an *ermB* gene.

## Taxonomic conclusions

The results of the phylogenetic analysis based on the 16S rRNA and *recN* gene sequences, and ANI analysis indicate that strains 29896^T^ (*S. suivaginalis* sp. nov.) and 29887^T^ (*S. iners* sp. nov.) represent novel species within the genus *Streptococcus,* and 29892^T^ (*S. iners* subsp. *hyiners* subsp. nov.) represents a novel subspecies of 29887^T^ (*S. iners* sp. nov.). Based on the host origin, phenotypic, genotypic and phylogenetic characteristics of these novel strains, we propose the names *Streptococcus suivaginalis* sp. nov., S*treptococcus iners* sp. nov. and *Streptococcus iners* subsp. *hyiners* subsp. nov. for the type strains 29896^T^, 29887^T^ and 29892^T^, respectively.

## Description of *Streptococcus suivaginalis* sp. nov.

*Streptococcus suivaginalis* (su.i.va.gi.na'lis. L. masc. or fem. n. *sus*, swine; L. fem. n. *vagina*, vagina; L. adj. suff. -*alis*, suffix denoting pertaining to; N.L. masc. adj. *suivaginalis*, associated with pig vagina).

Gram-stain positive, non-spore-forming and non-motile cocci, occurring in pairs and small clusters. Colonies on blood agar plates are small, circular and non-pigmented, approximately 1 mm in diameter and *α*-haemolytic at 37 °C. Catalase-negative and oxidase-negative. Cells grow well in THY +5% filtered heat-inactivated horse serum, TSB and THIO at 37 °C in ambient air or 5% CO_2_ atmosphere during 24–48 h. Cells can grow at temperatures between 30 and 42 °C under both ambient air and anaerobic conditions, with optimal growth observed between 35 and 38 °C, but do not grow in the presence of 6.5% NaCl. Using API Rapid ID32 Strep (bioMérieux), cells were able to produce acid from mannitol, lactose, glycogen, pullulan, maltose and sucrose, but not from methyl-*β*-d-glucopyranoside, tagatose, ribose, sorbitol, trehalose, raffinose, l-arabinose, d-arabitol, cyclodextrin, melibiose and melezitose. Cells were able to produce *β*-galactosidase and alanyl–pheylalanyl–proline–arylamidase, but no activity was detected for *β*-glucosidase, *β*-glucuronidase, *α*-galactosidase, alkaline phosphatase, pyroglutamic acid arylamidase, *N*-acetyl-*β*-glucosaminidase, glycyl–tryptophan arylamidase and *β*-mannosidase. Voges–Proskauer test was negative. Arginine, hippurate and urea were not hydrolysed. Cells tested negative for Lancefield group A, B, C, D, F or G.

The type strain, 29896^T^ (=NRRL B-65677^T^=NCTC 14941^T^), was isolated from a vaginal swab of a healthy pig during a routine veterinary physical exam. The full habitat is not known. The DNA G-C content of the type strain is 43.4 mol%. The DDBJ/ENA/GenBank accession numbers for the 16S rRNA gene sequence and chromosome are CP118733 and OR652396, respectively.

## Description of *Streptococcus iners* sp. nov.

*Streptococcus iners* (in'ers. L. masc. adj. *iners*, inert, inactive).

Gram-stain positive, non-spore-forming non-motile cocci occurring in pairs or chains usually 10 or more cells long. Good growth occurs on either TSA with 5% sheep blood or Columbia agar with 5% sheep blood. Colonies on blood agar plates are small, circular and non-pigmented, approximately 1 mm in diameter and *α*-haemolytic at 37 °C. Catalase-negative and oxidase-negative. Cells grow well in THY +5% filtered heat-inactivated horse serum, TSB and THIO at 37 °C in ambient air or 5% CO_2_ atmosphere during 24–48 h. Cells can grow at temperatures between 30 and 42 °C under both ambient air and anaerobic conditions, with optimal growth observed between 35 and 38 °C, but do not grow in the presence of 6.5% NaCl. Using API Rapid ID32 Strep (bioMérieux), cells were able to produce acid from trehalose and sucrose, but not from methyl-*β*-d-glucopyranoside, tagatose, ribose, mannitol, sorbitol, lactose, raffinose, l-arabinose, d-arabitol, cyclodextrin, glycogen, pullulan, maltose, melibiose and melezitose. Cells were able to produce *β*-glucuronidase and alanyl–pheylalanyl–proline–arylamidase, but no activity was detected for *β*-glucosidase, *β*-galactosidase, *α*-galactosidase, alkaline phosphatase, pyroglutamic acid arylamidase, *N*-acetyl-*β*-glucosaminidase, glycyl–tryptophan arylamidase and *β*-mannosidase. Voges–Proskauer test was negative. Arginine, hippurate and urea were not hydrolysed. Cells tested positive for Lancefield group G.

The type strain, 29887^T^ (=NRRL B-65675^T^=NCTC 14939^T^), was isolated from a vaginal swab of a healthy pig during a routine veterinary physical exam. The full habitat is not known. The DNA G-C content of the type strain is 42.1 mol%. The DDBJ/ENA/GenBank with the accession numbers for the 16S rRNA gene sequence, chromosome and plasmid are OR654109, CP118735 and CP118736, respectively.

The species contains one subspecies.

## Description of *Streptococcus iners* SUBSP. *hyiners* SUBSP. nov.

*Streptococcus iners* subsp. *hyiners* subsp. nov. (hy.in'ers. Gr. masc. or fem. n. *hys*, pig; L. masc. adj. *iners*, inert, inactive; N.L. masc. adj. *hyiners*, inactive bacterium derived from or related to swine).

Cells were seen occurring in pairs or small chains of three to six cells long. Strains were able to produce acid from mannitol, lactose, raffinose and maltose. Cells were not able to produce *β*-glucuronidase. Cells tested negative for Lancefield group A, B, C, D, F or G.

The type strain, 29892^T^, (=NRRL B-65676^T^=NCTC 14940^T^), was isolated from a vaginal swab of a healthy pig during a routine veterinary physical exam. The full habitat is not known. The DNA G-C content of the type strain is 42.5 mol%.

The DDBJ/ENA/GenBank accession numbers for the 16S rRNA gene sequence and chromosome are OR652397 and CP118734, respectively.

## supplementary material

10.1099/ijsem.0.006631 Uncited Table S1.

## Abbreviations

ANI: average nucleotide identity
dDDH: digital DNA–DNA hybridization
GBDP: genome blast distance phylogeny
MICs: minimum inhibitory concentrations
MLSb: macrolide/lincosamide/streptogramin
NJ: neighbour-joining
THIO: thioglycolate broth
THY: Todd Hewitt broth supplemented with 0.2% yeast extract
TSA: tryptic soy agar
TSB: tryptic soy broth
TYGS: Type (Strain) Genome Server
