# Supplementary material for: Streptococcus suivaginalis sp. nov., Streptococcus iners sp. nov. and Streptococcus iners subsp. hyiners subsp. nov. isolated from pigs
Source: Int J Syst Evol Microbiol. 2025 Jan 22;75(1):006631. doi: 10.1099/ijsem.0.006631 (PMC11753468; doi:10.1099/ijsem.0.006631)
Supplement: Uncited Table S1. [file ijsem-75-06631-s001.pdf]

Supplemental Table 1		
**RefSeq accession number**	**Locus Tag Prefix**	**Genbank Strain Name/designation**
NZ_CP118733	PXH68	29896[T] (*S. suivaginalis* sp. nov.) chromosome, complete genome
NZ_CP118735	PW252	29887[T] (*S. iners* sp. nov.) chromosome, complete genome
NZ_CP118734	PW220	29892[T] (*S. iners* subsp. *hyiners* subsp. nov.) chromosome, complete genome
GCF_000026745.1	SSUBM407	*S. suis* BM407[a]
GCF_000187585.1	HMPREF9180	*S. peroris* ATCC 700780[T]
GCF_000188015.2	STRIC	*S. ictaluri* 707-05[T]
GCF_000191105.1	HMPREF9390	*S. sanguinis* SK405 strain CCUG[T]
GCF_000221985.1	SPPN	*S. pseudopneumoniae* IS7493[a]
GCF_000300915.1	A0G	*S. iniae* 9117[a]
GCF_000377005.1	F602	*S. minor* DSM 17118[T]
GCF_000379985.1	A3GS	*S. caballi* DSM 19004[T]
GCF_000380005.1	A3GU	*S. didelphis* DSM 15616[T]
GCF_000380025.1	A3GW	*S. entericus* DSM 14446[T]
GCF_000380045.1	A3I1	*S. marimammalium* DSM 18627[T]
GCF_000380105.1	A3I7	*S. orisratti* DSM 15617[T]
GCF_000380125.1	A3I9	*S. ovis* DSM 16829[T]
GCF_000380145.1	A3IA	*S. thoraltensis* DSM 12221[T]
GCF_000420785.1	G574	*S. hyovaginalis* DSM 12219[T]
GCF_000423725.1	G573	*S. devriesei* DSM 19639[T]
GCF_000423745.1	G575	*S. plurextorum* DSM 22810[T]
GCF_000423765.1	G576	*S. porci* DSM 23759[T]
GCF_000425025.1	H346	*S. castoreus* DSM 17536[T]
GCF_000767835.1	SSIN	*S. sinensis* strain HKU4[T]
GCF_000960005.1	TZ90	*S. mitis* strain OT25[a]
GCF_001302265.1	AKK44	*S. phocae* strain ATCC 51973[T]
GCF_001375655.1	BN1356	*S. varani* strain FF10[T]
GCF_001412635.1	SanJ4211	*S. anginosus* strain J4211[a]
GCF_001431045.1	SCSH	*S. orisasini* strain SH06[a]
GCF_001552035.1	AMM49	*S. agalactiae* strain NGBS128[a]
GCF_001598035.1	A2G56	*S. halotolerans* strain HTS9[T]
GCF_001623565.1	A4H00	*S. marmotae* strain HTS5[T]
GCF_001642085.1	A0O21	*S. pantholopis* strain TA 26[T]
GCF_001657915.1	A7A96	*S. vestibularis* strain 22-06 S6[a]
GCF_001708305.1	BFM96	*S. himalayensis* strain HTS2[T]
GCF_001885095.1	A9Q68	*S. bovimastitidis* strain NZ1587[T]
GCF_001921845.1	BU202	*S. cuniculi* strain CCUG 65085[T]
GCF_001984705.1	BVE86	*S. azizii* strain 12-5291[T]
GCF_002076835.1	SPNHU17	*S. pneumoniae* strain Hu17[a]
GCF_002355215.1	SRT	*S. troglodytae* strain TKU 31[T]
GCF_002860805.1	CYK21	*S. macedonicus* strain UMB0733[a]
GCF_002887775.1	AT575	*S. penaeicida* strain CAIM 1838[T]
GCF_002900385.1	SPSF3K	*S. parauberis* strain SPOF3K[a]
GCF_002953735.1	C0J00	*S. pluranimalium* strain TH11417[T]
GCF_003086355.1	DDV21	*S. chenjunshii* strain Z15[T]
GCF_003143695.2	DB729	*S. halitosis* strain VT-4[T]
GCF_003172975.1	DK182	*S. sobrinus* strain 10919[T]
GCF_003438245.1	DXB95	*S. ilei* strain OM06-9[a]
GCF_003595525.1	CHF41	*S. respiraculi* strain HTS25[T]
GCF_003609975.1	SR187	*S. ruminantium* strain GUT-187[T]
GCF_003626515.1	D7D54	*S. chosunense* strain ChDC B353[T]
GCF_003627135.1	D7D50	*S. koreensis* strain KCOM 2890[T]
GCF_003627155.1	D7D53	*S. gwangjuense* strain KCOM 1679[T]
GCF_003686955.1	EAF07	*S. hillyeri* strain 28462[T]
GCF_003963555.1	EHW89	S. periodonticum strain KCOM 2412[T]
GCF_004353325.1	E2R31	*S. downii* strain CECT 9732[a]
GCF_004785935.1	E5S68	*S. rubneri* strain DSM 26920[T]

GCF_004843545.1	E8M05	*S. pasteurianus* strain WUSP067[a]
GCF_006385165.1	FJN11	*S. symci* strain C17[T]
GCF_006385785.1	FJR77	*S. shenyangsis* strain D19[T]
GCF_006385805.1	FJR71	*S. xiaochunlingii* strain E24[T]
GCF_007859195.1	FRX57	*S. cuniculipharyngis* strain CCUG 66496[T]
GCF_008803015.1	FY406	*S. ratti* strain ATCC 31377[a]
GCF_009738105.1	F5989	*S. mutans* strain NCH105[a]
GCF_009755785.1	GO995	*S. ruminicola* strain CNU_G3[a]
GCF_009767945.1	E5983	*S. danieliae* strain NM51_B2-22[a]
GCF_010993845.2	GE022	*S. canis* strain HL_77_2[a]
GCF_011421455.1	HBN43	*S. catagoni* strain 202-1/2017[T]
GCF_012396585.1	HF992	*S. ovuberis* strain CCUG 69612[T]
GCF_012843165.1	HHO37	*S. ratti* strain DSM 22768[T]
GCF_013267695.1	FOC63	*S. gallolyticus* strain FDAARGOS_755[a]
GCF_015689455.1	I2437	*S. equi* subsp. *zooepidemicus* strain SEZ33[a]
GCF_016127275.1	I6H76	*S. infantarius* strain FDAARGOS_1019[a]
GCF_016461705.1	JG537	*S. vicugnae* strain SL1232[T]
GCF_016481305.1	JHK62	*S. pacificus* strain CSL7591[T]
GCF_016724885.1	I6J14	*S. dysgalactiae* strain FDAARGOS_1157[a]
GCF_016908645.1	JOC28	*S. loxodontisalivarius* strain DSM 27382[T]
GCF_016908655.1	JOC31	*S. saliviloxodontae* strain DSM 27513[T]
GCF_017315345.1	JR342	*S. vaginalis* strain P1L01[T]
GCF_017883985.1	C4K46	*S. oricebi* strain CCUG 70868[a]
GCF_017884005.1	DHL47	*S. panodentis* strain CCUG 70867[a]
GCF_018127725.1	J4854	*S. lactarius* strain CCUG 66490[a]
GCF_018137985.1	INT76	*S. oriscaviae* strain HKU75[T]
GCF_019218685.1	KUA57	*S. vulneris* strain DM3B3[T]
GCF_019774635.1	K5J94	*S. halichoeri* strain Shali_VAS-CPH[a]
GCF_019929665.1	K1I61	*S. infantis* strain STn400[a]
GCF_021654455.1	L6410	*S. parasuis* strain SUT-286[T]
GCF_024170735.1	J2T51	*S. gallinaceus* strain 381D[a]
GCF_024346585.1	STYK	*S. toyakuensis* strain TP1632[T]
GCF_024742095.1	NW239	*S. zalophi* strain CSL10143-OR1[a]
GCF_027474505.1	O6R09	*S. alactolyticus* strain LGM[T]
GCF_900095845.1	BQ2679	S. timonensis strain Marseille-P2915[T]
GCF_900102715.1	BLR40	*S. equinus* strain pGA-7[a]
GCF_900104235.1	BLQ04	*S. henryi* strain A-4[a]
GCF_900187085.1	CKV83	*S. merionis* strain NCTC13788[T]
GCF_900459045.1	DYA48	*S. acidominimus* strain NCTC12957[T]
GCF_900459125.1	DYD51	*S. constellatus* strain NCTC11325[T]
GCF_900459175.1	DYE66	*S. downei* strain NCTC 11391[T]
GCF_900459215.1	DYC09	*S. criceti* strain NCTC12277[T]
GCF_900459355.1	DYA29	*S. parasanguinis* strain NCTC12854[a]
GCF_900459365.1	DYC10	*S. massiliensis* strain NCTC13765[T]
GCF_900459405.1	DYA54	*S. hyointestinalis* strain NCTC12224[T]
GCF_900459485.1	DYB47	*S. macacae* NCTC 11558[T]
GCF_900460135.1	DYA22	*S. uberis* strain NCTC4672[a]
GCF_900475025.1	DQL21	*S. ferus* strain NCTC12278[T]
GCF_900475035.1	DQM35	*S. pyogenes* strain NCTC12064[a]
GCF_900475445.1	DQM67	*S. cristatus* strain NCTC 12479[T]
GCF_900475675.1	DQN23	*S. lutetiensis* strain NCTC13774[a]
GCF_900475975.1	DQN42	*S. intermedius* strain NCTC11324[T]
GCF_900636505.1	EL100	*S. australis* strain NCTC3168[a]
GCF_900636885.1	EL133	*S. urinalis* strain NCTC13766 [T]
GCF_900637025.1	EL140	*S. oralis* strain NCTC 11427[T]
GCF_900637075.1	EL147	*S. pseudoporcinus* strain NCTC13786[T]
GCF_901544385.1	FGL23	*S. gordonii* strain NCTC10231[a]
GCF_901553735.1	FGL20	*S. porcinus* strain NCTC10925[a]

GCF_902858935.1	HV374	*S. salivarius* clinical isolate strain from anonymous[a]
GCF_903886475.1	H0506	*S. thermophilus* isolate STH_CIRM_65[a]
GCF_943193075.1	NFR56	*S. caecimuris* strain DSM 110150[T]
[a]NCBI RefSeq Genome or reference genome sequence.		
